# Clonal hematopoiesis associated with epigenetic aging and clinical outcomes

**DOI:** 10.1111/acel.13366

**Published:** 2021-05-29

**Authors:** Daniel Nachun, Ake T. Lu, Alexander G. Bick, Pradeep Natarajan, Joshua Weinstock, Mindy D. Szeto, Sekar Kathiresan, Goncalo Abecasis, Kent D. Taylor, Xiuqing Guo, Russ Tracy, Peter Durda, Yongmei Liu, Craig Johnson, Stephen S. Rich, David Van Den Berg, Cecilia Laurie, Tom Blackwell, George J. Papanicolaou, Adolfo Correa, Laura M. Raffield, Andrew D. Johnson, Joanne Murabito, JoAnn E. Manson, Pinkal Desai, Charles Kooperberg, Themistocles L. Assimes, Daniel Levy, Jerome I. Rotter, Alex P. Reiner, Eric A. Whitsel, James G. Wilson, Steve Horvath, Siddhartha Jaiswal, the NHLBI Trans‐Omics for Precision Medicine (TOPMed) Consortium

**Affiliations:** ^1^ Department of Pathology Stanford University School of Medicine Stanford CA USA; ^2^ Department of Human Genetics David Geffen School of Medicine University of California Los Angeles Los Angeles CA USA; ^3^ Department of Medicine Vanderbilt University School of Medicine Nashville TN USA; ^4^ Department of Medicine Massachusetts General Hospital Boston MA USA; ^5^ Broad Institute Cambridge MA USA; ^6^ Department of Biostatistics University of Michigan School of Public Health Ann Arbor MI USA; ^7^ Division of Biomedical Informatics and Personalized Medicine Department of Medicine University of Colorado Anschutz Medical Campus Aurora CO USA; ^8^ Department of Pediatrics The Institute for Translational Genomics and Population Sciences The Lundquist Institute for Biomedical Innovation at Harbor‐UCLA Medical Center Torrance CA USA; ^9^ Department of Pathology and Laboratory Medicine Larner College of Medicine University of Vermont Burlington VT USA; ^10^ Department of Medicine Duke University Medical Center Durham NC USA; ^11^ Department of Biostatistics School of Public Health University of Washington Seattle WA USA; ^12^ Center for Public Health Genomics University of Virginia Charlottesville VA USA; ^13^ Department of Clinical Preventative Medicine Keck School of Medicine University of Southern California Los Angeles CA USA; ^14^ Division of Cardiovascular Sciences National Heart, Lung, and Blood Institute Bethesda MD USA; ^15^ Department of Medicine University of Mississippi Medical Center Jackson MS USA; ^16^ Department of Genetics University of North Carolina School of Medicine Chapel Hill NC USA; ^17^ National Heart Lung and Blood Institute Framingham MA USA; ^18^ Department of Medicine Boston University School of Medicine Boston MA USA; ^19^ Department of Medicine Brigham and Women's Hospital Harvard Medical School Boston MA USA; ^20^ Department of Hematology/Oncology Joan & Sanford I. Weill Medical College of Cornell University New York NY USA; ^21^ Public Health Sciences Division Fred Hutchinson Cancer Research Center Seattle WA USA; ^22^ Department of Medicine Stanford University School of Medicine Stanford CA USA; ^23^ Department of Epidemiology School of Public Health University of Washington Seattle WA USA; ^24^ Department of Medicine University of North Carolina Chapel Hill NC USA; ^25^ Department of Epidemiology Gillings School of Global Public Health Chapel Hill NC USA; ^26^ Department of Cardiology Beth Israel Deaconess Medical Center Boston MA USA; ^27^ Department of Physiology and Biophysics University of Mississippi Medical Center Jackson MS USA; ^28^ Institute for Stem Cell Biology and Regenerative Medicine Stanford University School of Medicine Stanford CA USA

**Keywords:** clonal hematopoiesis, epigenomics, heart disease

## Abstract

Clonal hematopoiesis of indeterminate potential (CHIP) is a common precursor state for blood cancers that most frequently occurs due to mutations in the DNA‐methylation modifying enzymes *DNMT3A* or *TET2*. We used DNA‐methylation array and whole‐genome sequencing data from four cohorts together comprising 5522 persons to study the association between CHIP, epigenetic clocks, and health outcomes. CHIP was strongly associated with epigenetic age acceleration, defined as the residual after regressing epigenetic clock age on chronological age, in several clocks, ranging from 1.31 years (GrimAge, *p* < 8.6 × 10^−7^) to 3.08 years (EEAA, *p* < 3.7 × 10^−18^). Mutations in most CHIP genes except DNA‐damage response genes were associated with increases in several measures of age acceleration. CHIP carriers with mutations in multiple genes had the largest increases in age acceleration and decrease in estimated telomere length. Finally, we found that ~40% of CHIP carriers had acceleration >0 in both Hannum and GrimAge (referred to as AgeAccelHG+). This group was at high risk of all‐cause mortality (hazard ratio 2.90, *p* < 4.1 × 10^−8^) and coronary heart disease (CHD) (hazard ratio 3.24, *p* < 9.3 × 10^−6^) compared to those who were CHIP−/AgeAccelHG−. In contrast, the other ~60% of CHIP carriers who were AgeAccelHG− were not at increased risk of these outcomes. In summary, CHIP is strongly linked to age acceleration in multiple clocks, and the combination of CHIP and epigenetic aging may be used to identify a population at high risk for adverse outcomes and who may be a target for clinical interventions.

## Graphical abstract

Clonal hematopoiesis of indeterminate potential (CHIP) and epigenetic age acceleration are the two important aging phenomenon associated with adverse clinical outcomes. We found that mutations in most CHIP genes were associated with increased age acceleration in multiple epigenetic clocks. Individuals with CHIP and age acceleration had a greatly increased risk of mortality and coronary heart disease compared to individuals with only CHIP or age acceleration.

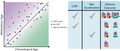

**Abbreviations**
BMI: body mass index
CHD: coronary heart disease
CHIP: clonal hematopoiesis of indeterminate potential
DDR: DNA damage response
EEAA: extrinsic epigenetic age acceleration
FHS: Framingham Heart Study
HDL: high density lipoprotein
HSC: hematopoietic stem cell
hsCRP: high‐sensitivity C‐reactive protein
IEAA: intrinsic epigenetic age acceleration
JHS: Jackson Heart Study
LDL: low density lipoprotein
LTL: leukocyte telomere length
MESA: Multi‐ethnic Study of Atherosclerosis
SNP: single nucleotide polymorphism
TOPMed: Trans‐omics for Precision Medicine
VAF: variant allele fraction
WGS: whole genome sequencing
WHI: Women's Health Initiative

## 1 INTRODUCTION

Aging is inextricably associated with an increase in the number of somatic mutations, and this process is believed to be central to the development of cancer (Blokzijl et al., 2016; Hoang et al., 2016; Martincorena & Campbell, 2015; Risques & Kennedy, 2018; Welch et al., 2012). Clonal hematopoiesis of indeterminate potential (CHIP) (Jaiswal et al., 2014) is defined by the presence of a cancer‐associated somatic mutation in the blood cells of people without a blood cancer or other known clonal disorder. CHIP originates when hematopoietic stem cells (HSCs) acquire a random mutation, usually in an epigenetic factor, that results in increased clone fitness (Jaiswal & Ebert, 2019). CHIP is strongly associated with age, and carriers of these mutations have an increased risk for developing blood cancers, but also coronary heart disease (CHD) and all‐cause mortality (Jaiswal et al., 2014, 2017). In addition to age, CHIP has been found to occur at a higher prevalence in males (Jaiswal et al., 2014) and a lower prevalence in people of self‐reported Hispanic and East Asian ancestry compared to Europeans (Bick, Weinstock, et al., 2020; Jaiswal et al., 2014). The association of CHIP and heart disease may result from enhanced inflammatory gene expression in mutant macrophages within atherosclerotic plaques (Bick, Pirruccello, et al., 2020; Fuster et al., 2017; Jaiswal et al., 2017), demonstrating that at least some of these mutations cause dysfunction of immune cells and affect phenotypes apart from cancer.

The availability of DNA‐methylation data from large epidemiological cohorts has advanced our understanding of epigenetic aging in recent years. Several “methylation clocks” have been developed (Hannum et al., 2013; Horvath, 2013; Horvath et al., 2018; Levine et al., 2018; Lu, Quach, et al., 2019) that use methylation state at a subset of CpGs to predict chronological age with high accuracy in healthy individuals. “Age acceleration” results when predicted methylation age is greater than chronological age and associates with increased risk of CHD (Levine et al., 2018; Lu, Quach, et al., 2019; Perna et al., 2016) and all‐cause mortality (Chen et al., 2016; Christiansen et al., 2016; Levine et al., 2018; Lu, Quach, et al., 2019, p. 201; Marioni et al., 2015; Perna et al., 2016). Similar to prior studies (Horvath & Raj, 2018), we defined age acceleration as the residual of a linear model of a clock estimate regressed against chronological age. By definition, this measure is not correlated with chronological age and a positive (or negative) value indicates that the clock age is higher (or lower) than expected based on chronological age. The factors underlying epigenetic age acceleration are incompletely understood. Recent work has noted that two distinct categories of epigenetic clocks, intrinsic and extrinsic, which are believed to capture different aspects of aging. Intrinsic aging is independent of cell type and may be partly driven by the number of times a cell has divided (Lu et al., 2018), while extrinsic aging, is associated with changes of cell type composition in blood (Horvath et al., 2016), and maybe influenced by environmental factors (Levine et al., 2018; Lu, Quach, et al., 2019). The Horvath and IEAA clocks reflect intrinsic aging, whereas the Hannum, EEAA, PhenoAge, and GrimAge clocks are measures of extrinsic aging (Table 1). GrimAge and PhenoAge were also trained to be predictors of mortality (Levine et al., 2018; Lu, Quach, et al., 2019). In addition, several DNA methylation‐based predictors of other aging‐related phenotypes have recently been developed to improve mortality prediction, such as surrogate biomarkers for plasma protein levels (adrenomedullin, beta‐2‐microglobulin, cystatin C, leptin, plasminogen activator inhibitor 1, tissue inhibitor matrix metalloproteinase 1) (Lu, Quach, et al., 2019), smoking pack years (Lu, Quach, et al., 2019), and telomere length (Lu, Seeboth, et al., 2019).

**TABLE 1:** Summary of epigenetic clocks used in the study

Clock	Type	Tissue	Outcome	Publication	Notes
Horvath	Intrinsic	Multiple	Chronological age	Horvath (2013)	Inaccessible tissues primarily from tissue‐adjacent normal samples in The Cancer Genome Atlas (see publication)
IEAA	Intrinsic	Multiple	Chronological age	Quach et al. (2017)	Uses same CpGs as Horvath clock, but reweighted as described in Quach et al. to minimize influence of cell composition
Hannum	Extrinsic	Whole blood	Chronological age	Hannum et al. (2013)	Highly correlated with aging‐related changes in blood cell composition
EEAA	Extrinsic	Whole blood	Chronological age	Quach et al. (2017)	Uses same CpGs as Hannum clock, but reweighted as described in Quach et al. to maximize influence of cell composition
SkinAndBloodClock	Intrinsic	Whole blood, fibroblasts	Chronological age	Horvath et al. (2018)	Created to address poor age prediction in Horvath clock in skin and whole blood
PhenoAge	Extrinsic	Whole blood	Time to death	Levine et al. (2018)	PhenoAge is measure of mortality risk derived from National Health and Nutrition Examination Survey using the following markers: albumin, creatinine, serum glucose, log C‐reactive protein, lymphocyte percent, mean red cell volume, red cell distribution width, alkaline phosphatase, white blood cell count, and age (see publication for details)
GrimAge	Extrinsic	Whole blood	Time to death	Lu, Quach, et al. (2019)	Methylation is used to predict eight surrogate biomarkers: Adrenomedullin (ADM), Beta‐2‐Microglobulin (B2M), Cystatin C, Growth Differentiation Factor 15 (GDF15), Leptin, Serpin Family E Member 1 (SERPINE/PAI1), TIMP Metalloproteinase Inhibitor 1 (TIMP1), smoking pack‐years (PACKYRS). The predicted values of those biomarkers are used to predict mortality (see publication for details)

Abbreviations: EEAA, extrinsic epigenetic age acceleration; IEAA, intrinsic epigenetic age acceleration.

We hypothesized that CHIP may be an acquired genetic factor associated with epigenetic age acceleration. Here, we use whole‐genome sequencing (WGS) and DNA‐methylation array data from several cohorts within the Trans‐omics for Precision Medicine (TOPMed) program to test the hypothesis that CHIP is linked to epigenetic age acceleration. We find that CHIP is strongly associated with age acceleration in several clocks. We further assess whether there are gene‐specific associations of CHIP with epigenetic age and methylation‐estimated telomere length. Finally, we test whether the combination of CHIP status and epigenetic age can be used to identify the group at highest risk for adverse outcomes.

## 2 RESULTS

### 2.1 Association between CHIP and epigenetic age acceleration in several clocks

We used WGS data obtained from whole blood DNA for several large cohorts within TOPMed, including the Framingham Heart Study (FHS), the Jackson Heart Study (JHS), the Women's Health Initiative (WHI), and the Multi‐Ethnic Study of Atherosclerosis (MESA), to identify CHIP as previously described (Bick, Pirruccello, et al., 2020; Bick, Weinstock, et al., 2020) (see Table S1 for a demographic summary of cohorts). The populations assayed for methylation were an unbiased selection from within FHS and JHS, while the WHI TOPMed samples were over‐sampled for incident stroke and venous thromboembolism. The BA23 subset of WHI was a CHD case/control study. Importantly, the blood draw used for methylation array analysis was the same as that used for WGS in FHS, JHS and MESA, and in WHI, only persons for whom the blood draw for the WGS was within 3 years of the draw for methylation were included. After adjusting age acceleration residuals for sex, self‐reported ancestry, and cohort, 5522 individuals, including 319 CHIP carriers, from the four cohorts were assessed for seven different aging measures: DNAmAge (Horvath) (Horvath, 2013), DNAmHannum (Hannum) (Hannum et al., 2013), DNAmPhenoAge (PhenoAge) (Levine et al., 2018), DNAmSkinClock (SkinBloodClock) (Horvath et al., 2018), DNAmGrimAge (GrimAge) (Lu, Quach, et al., 2019), intrinsic epigenetic age acceleration (IEAA) (Lu et al., 2018) and extrinsic epigenetic age acceleration (EEAA) (Lu et al., 2018), and a methylation‐based estimate of telomere length (DNAmTL) (see Methods). The effects of CHIP were assessed overall (any CHIP mutation), as well as at the level of specific classes of CHIP mutations (see Methods).

**FIGURE 1:**
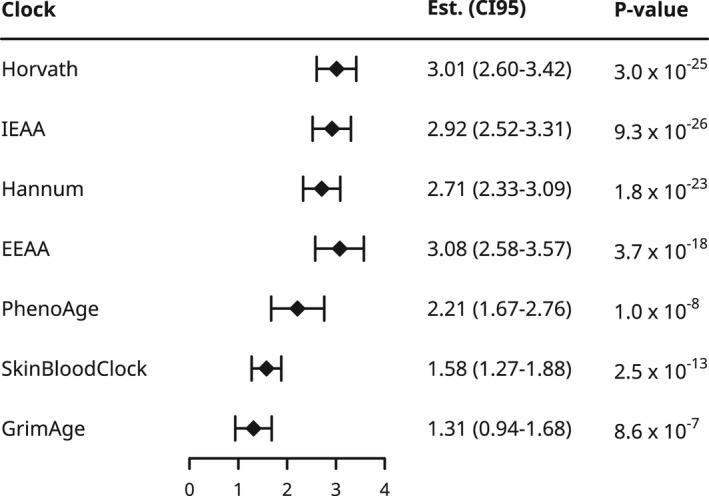
CHIP is associated with increased age acceleration. Forest plot of the effect sizes and confidence intervals for the effect of CHIP on age acceleration estimate from seven methylation clocks

Consistent with previous results, carriers of CHIP were significantly older than non‐carriers (+7.23 ± 0.61 years, *p* < 1.13 × 10^−31^, Figures S1 and S2), and the prevalence of CHIP reached >20% in those over 80 years (Figure S1). We then tested whether age acceleration residuals from several clocks bore any association to CHIP (Figure 1). Similar to the results of Robertson et al. (2019), CHIP was most strongly associated with intrinsic age acceleration (Horvath: 3.01 years, *p* < 3.0 × 10^−25^; IEAA: 2.92 years, *p* < 9.3 × 10^−26^). Due to our larger sample size, we also observed strong associations between CHIP and extrinsic age acceleration (Hannum clock: 2.71 years, *p* < 1.8 × 10^−23^; EEAA: 3.08 years, *p* < 3.7 × 10^−18^), as well as PhenoAge (2.21 years, *p* < 1.0 × 10^−8^), SkinBloodClock (1.58 years, *p* < 2.5 × 10^−13^), and GrimAge (1.31 years, *p* < 8.6 × 10^−7^). We also found that the number of driver mutations was associated with a stepwise increase in age acceleration for several clocks, and this relationship was strongest for Hannum and EEAA (Table S2).

We also found modest associations between CHIP and several epigenetic surrogate markers of plasma proteins as well as blood counts (Table S3A,B), and between clock estimates and variant allele fraction (VAF), which is an approximation of clone size (Table S4). Methylation data can also be used to estimate a surrogate marker of leukocyte telomere length (LTL), DNAmTL (Lu, Seeboth, et al., 2019). CHIP was associated with reduced predicted age‐adjusted DNAmTL in CHIP overall (−0.06, *p* < 1.2 × 10^−8^), as well as several mutation classes (Figure S3A). An increasing number of mutations was associated with a decrease in predicted DNAmTL (2 mut. vs. 1: −0.174, *p* < 8.0 × 10^−7^; >2 mut. vs. 2: −0.404, *p* < 1.1 × 10^−5^, Figure S3B,C).

### 2.2 Gene‐specific associations of CHIP with epigenetic age acceleration

Clonal hematopoiesis of indeterminate potential most commonly occurs due to mutations in genes coding for the DNA methylation‐altering enzymes *DNMT3A* and *TET2*, but can also arise due to mutations in *ASXL1*, *JAK2*, splicing factors, and DNA‐damage response (DDR) genes. Accordingly, we examined the associations of mutations in specific CHIP genes with age acceleration (Table 2). In all clocks, the direction of association for *DNMT3A* and *TET2* mutations was the same, although those with *TET2* mutations had significantly greater age acceleration than those with *DNMT3A* mutations for Hannum (2.10 years, *p* < 0.0012) and EEAA (2.32 years, *p* < 0.0063), but not other clocks. We also performed differential methylation analysis to assess whether mutations in the DNA‐methylation modifying enzymes *DNMT3A* and *TET2* had divergent effects at the clock CpGs. Mutations in both genes primarily resulted in hypomethylation although a small number of CpGs showed hypermethylation in *TET2* (Figure S4A,B). We also observed at the clock CpGs that the M‐values (a log‐transformed measure of the percent methylation at each site) in persons with *DNMT3A* and *TET2* mutations were highly correlated (Figure S4C), indicating that the methylation state of persons with the two mutations is largely similar, despite their opposing enzymatic effects.

**TABLE 2:** CHIP mutations in specific classes of genes have largely consistent effects on age acceleration

Class	Horvath		IEAA		Hannum		EEAA		SkinBloodClock		PhenoAge		GrimAge	
	Est. (SE)	*p*‐value	Est. (SE)	*p*‐value	Est. (SE)	*p*‐value	Est. (SE)	*p*‐value	Est. (SE)	*p*‐value	Est. (SE)	*p*‐value	Est. (SE)	*p*‐value
All	3.01 (0.27)	3.0 × 10^−25^	2.92 (0.26)	9.30 × 10^−26^	2.71 (0.26)	1.80 × 10^−23^	3.08 (0.33)	3.70 × 10^−18^	1.58 (0.20)	2.50 × 10^−13^	2.21 (0.36)	1.00 × 10^−08^	1.31 (0.25)	8.60 × 10^−07^
*DNMT3A*	2.58 (0.38)	2.20 × 10^−10^	2.72 (0.36)	2.10 × 10^−12^	1.76 (0.35)	5.70 × 10^−06^	1.75 (0.46)	6.80 × 10^−04^	1.44 (0.28)	1.80 × 10^−06^	2.16 (0.51)	5.80 × 10^−05^	0.61 (0.35)	0.123
*TET2*	2.58 (0.59)	4.80 × 10^−05^	2.47 (0.57)	4.90 × 10^−05^	3.86 (0.55)	2.10 × 10^−11^	4.07 (0.72)	7.20 × 10^−08^	0.91 (0.44)	0.06	1.31 (0.79)	0.135	0.99 (0.55)	0.093
Multiple	7.43 (0.93)	5.60 × 10^−15^	6.77 (0.89)	1.10 × 10^−13^	8.36 (0.86)	3.0 × 10^−21^	10.97 (1.13)	2.50 × 10^−21^	5.01 (0.69)	1.00 × 10^−12^	6.35 (1.24)	5.30 × 10^−07^	4.85 (0.85)	2.40 × 10^−08^
DDR	0.21 (1.06)	0.962	−0.21 (1.01)	0.717	0.31 (0.98)	0.871	1.43 (1.29)	0.327	−0.26 (0.79)	0.66	0.63 (1.41)	0.718	−0.27 (0.97)	0.723
*JAK2*	3.80 (1.67)	0.029	1.37 (1.60)	0.448	5.88 (1.56)	2.30 × 10^−04^	6.21 (2.04)	0.003	4.31 (1.24)	6.70 × 10^−04^	10.01 (2.23)	9.70 × 10^−06^	3.46 (1.54)	0.028
*ASXL1/2*	2.86 (1.06)	0.011	2.75 (1.01)	0.011	1.46 (0.98)	0.183	1.87 (1.29)	0.188	0.44 (0.79)	0.652	−0.55 (1.41)	0.634	3.11 (0.97)	0.002
Splicing factor	5.02 (1.57)	0.002	4.88 (1.51)	0.002	2.70 (1.47)	0.082	2.41 (1.92)	0.242	2.36 (1.17)	0.052	2.46 (2.11)	0.267	2.37 (1.45)	0.112
Other	4.20 (1.31)	0.002	4.40 (1.26)	7.30 × 10^−04^	0.98 (1.22)	0.497	1.68 (1.60)	0.345	1.99 (0.97)	0.05	0.73 (1.75)	0.726	1.95 (1.21)	0.12

Note

Table with effect sizes, standard errors, and *p*‐values for eight different classes of CHIP mutations. “Multiple” means mutations in multiple genes. “DDR” refers to mutations in the DNA damage response genes *TP53*, *PPM1D*, and *BRCC3*. “Splicing factor” are mutations in *SF3B1*, *SRSF2*, *U2AF1*, *ZRSR2*, and *PRPF8*. “Other” refers to mutations in all other genes not listed.

Persons with mutations in multiple genes had the largest increases in age acceleration across all clocks except PhenoAge, consistent with our observation that age acceleration increases with the number of mutations. Conversely, no increase in age acceleration was observed in persons with mutations in DDR genes (*TP53*, *PPM1D, BRCC3*), which is consistent with the lack of association with age acceleration observed for the same mutations in cancer tissue samples (Horvath, 2013). Although we had only eight individuals with *JAK2* mutations in our cohort, these mutations showed an exceptionally strong association for a single mutation in several clocks, the most extreme example being PhenoAge (10.01 years, *p* < 9.7 × 10^−6^). The PhenoAge clock was trained to predict a composite measure of mortality risk which includes several hematological variables such as white blood cell count, white blood cell differential, and several red blood cell parameters which may be abnormal in myeloproliferative neoplasm, a hematological malignancy which is strongly associated with *JAK2* mutations. CHIP overall was nominally associated with estimated pack years of smoking (DNAmPACKYRS), but only mutations in *ASXL1* were significantly associated with this measure in a gene‐specific analysis (7.54 pack years, *p* < 0.002), a finding that is in accordance with a recent report (Bolton et al., 2020) (Table S3).

### 2.3 Association of CHIP and epigenetic age acceleration with clinical outcomes

**FIGURE 2:**
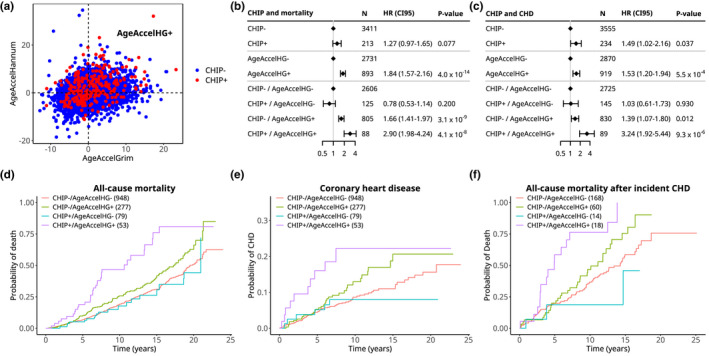
CHIP and epigenetic age acceleration identify persons at high risk of all‐cause mortality and development of coronary heart disease (CHD). (a) Scatterplot of correlation between AgeAccelGrim and AgeAccelHannum in all cohorts. (b, c) Forest plots showing hazard ratios, confidence intervals, and *p*‐values for Cox proportional hazard models of all‐cause mortality (b) and development of CHD (c) in persons from FHS, JHS, and WHI. All models included chronological age, race, low‐density lipoprotein cholesterol, high‐density lipoprotein cholesterol, triglycerides, systolic blood pressure, type 2 diabetes status and smoking status as covariates. Top two sections show the overall effect size of CHIP and age acceleration and bottom section shows effect sizes based on dividing persons into four groups based upon presence of CHIP and age acceleration. The results in c are a meta‐analysis of events in FHS, JHS, WHI EMPC (unselected for CHD), and WHI BA23 (case‐control study for CHD). (d, e) Cumulative incidence plots of death (d) and CHD (e) in persons divided into groups by the presence of CHIP (CHIP+/CHIP−) and age acceleration (AgeAccelHG+/AgeAccelHG−). The numbers in parentheses indicate the number of persons in each group for these analyses. Only persons over 65 and free of CHD at baseline were used in d and e, while all persons were used for b and c. (f) Cumulative incidence plot of death in persons with incident CHD after age 70. Individuals who died less than 30 days after CHD were excluded

Several previous studies have linked both CHIP (Jaiswal et al., 2014, 2017) and age acceleration in some clocks (Levine et al., 2018; Lu, Quach, et al., 2019) to increased risk of adverse clinical outcomes, in particular all‐cause mortality and CHD. We asked whether the combination of CHIP and age acceleration could further stratify carriers of CHIP into high‐risk and low‐risk groups for these outcomes using Cox proportional hazards models adjusted for chronological age at blood draw, low‐density lipoprotein cholesterol, high‐density lipoprotein cholesterol, triglycerides, systolic blood pressure, type 2 diabetes status, smoking status, and self‐reported ancestry in 4088 persons from JHS, FHS, and WHI (Figure 2B,C). In FHS, JHS, and WHI EMPC, which are unselected for CHD, there were 720 deaths (74 in CHIP carriers) out of 3624 participants (213 CHIP carriers) and 212 cases of incident CHD (22 in CHIP carriers) out of 3331 participants (192 CHIP carriers) after excluding those with CHD prevalent to time of blood draw. In WHI BA23, which was a case‐control study for CHD, there were 168 cases of incident CHD (18 in CHIP carriers) in 458 total participants (42 CHIP carriers).

We defined a person to have “age acceleration” (AgeAccel) for a clock if their values for an age acceleration residual exceeded zero after adjustment for age at blood draw, sex, self‐reported ancestry, and study cohort. We then tested the interaction between this dichotomous variable and CHIP status in predicting mortality in each of the seven clocks using Cox models. As shown in Table S5, we found that the most significant interactions were for the Hannum and GrimAge clocks, although neither reached Bonferroni‐corrected statistical significance. Though both the Hannum and GrimAge clocks were predictive of time to death or CHD in previous studies (Lu, Quach, et al., 2019; Marioni et al., 2015; Perna et al., 2016), they were trained on different outcomes (age for Hannum versus time to death for GrimAge), and are not strongly correlated in our dataset (bicor = 0.242, *R* ^2^ = 0.058, Figure 2A). Therefore, we reasoned that a combined measure incorporating age acceleration in *both* Hannum and GrimAge would better stratify high‐ and low‐risk groups because each clock provides orthogonal information. By this combined measure (henceforth referred to as AgeAccelHG), 102/255 (40%) of CHIP carriers were AgeAccelHG+ (age acceleration residual >0 for both Hannum and GrimAge), compared to 922/3833 (24%) persons without CHIP. Considered individually in separate models, CHIP and AgeAccelHG were each associated with a modest increase in risk of all‐cause mortality (CHIP: HR 1.27, *p* < 0.077; AgeAccelHG: HR 1.84, *p* < 4.0 × 10^−14^), consistent with previous findings. When we modeled the interaction of CHIP with AgeAccelHG for all‐cause mortality, we found a significant interaction effect (CHIP main effect: coefficient = −0.25, *p* < 0.20; AgeAccelHG main effect: coefficient = 0.51, *p* < 3.08 × 10^−9^; interaction: coefficient = 0.80, *p* < 3.74 × 10^−3^), which remained significant after Bonferroni correction for eight tests.

To validate this finding, we sought replication in an independent cohort, the BA23 subset of WHI, which was not used in the above mortality analysis (Horvath et al., 2016). When we modeled the interaction of CHIP with AgeAccelHG for CHD in BA23, the interaction term was again significant (CHIP main effect: coefficient = −0.24, *p* < 0.60; AgeAccelHG main effect: coefficient = 0.24, *p* < 0.35; interaction: coefficient = 1.72, *p* < 0.01).

Having demonstrated a significant statistical interaction between CHIP and AgeAccelHG for clinical outcomes, we combined these two variables into a single, 4‐factor variable for further modeling. For CHD, we included incident events in FHS, JHS, and WHI EMPC together with WHI BA23 as a meta‐analysis. Persons who were CHIP+/AgeAccelHG+ had much greater risk of all‐cause mortality (HR 2.90, *p* < 4.1 × 10^−8^) and CHD (HR 3.24, *p* < 9.3 × 10^−6^) compared to those who were CHIP−/AgeAccelHG−. Those who were CHIP−/AgeAccelHG+ had a more modest increase in risk of all‐cause mortality (HR 1.66, *p* < 3.1 × 10^−9^), and CHD (HR 1.39, *p* < 0.012) compared to those who were CHIP−/AgeAccelHG−. In contrast, those who were CHIP+/AgeAccelHG− did not have elevated risk of either all‐cause mortality (HR 0.78, *p* < 0.20) or CHD (HR 1.03, *p* < 0.93) compared to those who were CHIP−/AgeAccelHG− (Figure 2B,C). We also fitted contrasts to estimate the hazard ratios for all‐cause mortality and CHD for CHIP only in persons with AgeAccelHG+ and AgeAccelHG+ only in persons with CHIP, in both cases finding the associations to be significant (Figure S5).

We also asked if there were gene‐level differences in risk of these outcomes. We had insufficient sample size to assess either mortality or CHD individually, so we combined the two into a composite outcome. Being AgeAccelHG+ increased the risk of the composite outcome for those with *TET2* mutations relative to those who were CHIP−/AgeAccelHG− (*TET2* mutated+/AgeAccelHG+: HR = 3.88, *p* < 1.6 × 10^−6^; *TET2* mutated+/AgeAccelHG−: HR = 1.14, *p* < 0.66; *p* for interaction < 0.065) to a greater degree than those with *DNMT3A* mutations (*DNMT3A* mutated+/AgeAccelHG+: HR = 1.99, *p* < 0.028; *DNMT3A* mutated+/AgeAccelHG−: HR = 0.68, *p* < 0.079; *p* for interaction < 0.11) or other non‐DDR mutations (other mutation+/AgeAccelHG+: HR = 2.88, *p* < 1.1 × 10^−5^; other mutation+/AgeAccelHG−: HR = 1.00, *p* < 1; *p* for interaction < 0.19).

To illustrate absolute risks among those with both CHIP and AgeAccelHG, we determined the cumulative incidence of all‐cause mortality and CHD in persons from FHS, JHS, and WHI EMPC aged 65 or older at blood draw who did not have prevalent CHD (Figure 2D,E). Those who were CHIP+/AgeAccelHG+ had a cumulative incidence of all‐cause mortality of 46.6% by 10 years and a cumulative incidence of CHD of 22.2% by 10 years. In contrast, the other three groups had substantially lower 10‐year cumulative incidence of all‐cause mortality (CHIP+/AgeAccelHG− 17.7%, CHIP−/AgeAccelHG+ 25.8%, CHIP−/AgeAccelHG− 19.2%) and CHD (CHIP+/AgeAccelHG− 7.98%, CHIP−/AgeAccelHG+ 13.0%, CHIP−/AgeAccelHG− 8.66%).

Our data permitted us to also ask whether there was an association of CHIP and AgeAccelHG to time to death in those who had a first CHD event, a subgroup that is often the target of clinical interventions. We restricted our analysis to individuals who had a first CHD event after age 70 and, if they died, did so more than 30 days after the CHD event. We found a significant interaction between CHIP and AgeAccelHG for all‐cause mortality after CHD (*p* < 0.036). Persons who were CHIP+/AgeAccelHG+ showed significant increase in risk of all‐cause mortality (HR = 3.16, *p* < 1.16 × 10^−5^), while those who were CHIP+/AgeAccelHG− (HR = 0.462, *p* < 0.27) or CHIP−/AgeAccelHG+ (HR = 1.40, *p* < 0.13) showed no significant increase. The 5‐year cumulative incidence of death after CHD for those who were CHIP+/AgeAccelHG+ was 58.5%, while for all other groups it was substantially lower (CHIP+/AgeAccelHG− 18.8%, CHIP−/AgeAccelHG+ 20.0%, CHIP−/AgeAccelHG− 19.8%, Figure 2F).

Given the previous findings linking both CHIP (Jaiswal et al., 2017) and extrinsic epigenetic aging (Horvath et al., 2016; Levine et al., 2018; Lu, Quach, et al., 2019) to inflammation, we asked whether plasma levels of the inflammation marker high‐sensitivity C‐reactive protein (hs‐CRP) showed any evidence of interaction with CHIP for all‐cause mortality or CHD. We found evidence for a main effect of hs‐CRP on risk for all‐cause mortality, but not for an interaction with CHIP (CHIP main effect: coefficient = 0.22, *p* < 0.22; log (hs‐CRP) main effect: coefficient = 0.09, *p* < 1.01 × 10^−3^; interaction: coefficient = 0.076, *p* < 0.29). For CHD, no effect of hs‐CRP was observed (CHIP main effect: coefficient = 0.23 *p* < 0.49; log (hs‐CRP) main effect: coefficient = 0.01, *p* < 0.90; interaction: coefficient = −0.3, *p* < 0.82). We also stratified our cohort into eight groups based upon CHIP status, AgeAccelHG status, and whether hs‐CRP levels were above 2 mg/L, an established clinical cutoff. Individuals with CHIP and AgeAccelHG showed a similar risk of all‐cause mortality and CHD regardless of whether they had high or low hs‐CRP levels (Figure S5E,F). These results indicate that hs‐CRP is a poor discriminator of risk in CHIP carriers, unlike AgeAccelHG.

A coding SNP in *IL6R* (rs2228145), which results in Asp358Ala, was previously found to attenuate the increased risk for mortality and CHD associated with CHIP (Bick, Pirruccello, et al., 2020; Bick, Weinstock, et al., 2020). Here, the interaction between CHIP status and alternate allele count at rs2228145 was not significant for either all‐cause mortality (CHIP main effect: coefficient = 0.27, *p* < 0.158; rs2228145 main effect: coefficient = −0.082 per alternate allele, *p* < 0.21; interaction: coefficient = −0.044 per alternate allele, *p* < 0.82) or CHD (CHIP main effect: coefficient = 0.23, *p* < 0.36; rs2228145 main effect: coefficient = −0.16 per alternate allele, *p* < 0.08; interaction: coefficient = 0.25 per alternate allele, *p* < 0.36). There were also no significant interactions between rs2228145 genotype and the combined CHIP/AgeAccelHG variable (Figure S5C,D). These results indicate that *IL6R* genotype is a poor discriminator of risk in CHIP carriers in this dataset, unlike AgeAccelHG. However, we did find differences based on which gene was mutated. Those who were *TET2*‐CHIP+/AgeAccelHG+ and with no alternate alleles of rs2228145 (*IL6R*WT) had the highest risk for the composite mortality/CHD outcome relative to the referent group of CHIP−/AgeAccelHG−/*IL6R*WT (HR =11.3, *p* < 2.4 × 10^−21^, Figure S6). Those who were *TET2*‐CHIP+/AgeAccelHG+ but carried 1 or 2 alternate alleles of rs2228145 (*IL6R*Mut) had substantially lower risk (HR = 1.91 compared to the same referent group, *p* < 0.066; coefficient for interaction = −1.12 per alternate allele, *p* for interaction < 9.6 × 10^−7^, Figure S6). There was no significant difference in risk based on rs2228145 genotype in those who were *TET2*‐CHIP+/AgeAccelHG−. We also did not find significant differences in risk of death/CHD by rs2228145 genotype in *DNMT3A*‐CHIP or CHIP with other non‐DDR mutations regardless of AgeAccelHG status.

## 3 DISCUSSION

The results presented here permit us to draw several conclusions. First, it is clear that CHIP is strongly associated with epigenetic aging in several clocks. Consistent with the results from Robertson et al. (2019), we find the strongest associations to be with the intrinsic clocks, Horvath and IEAA. This could reflect a shared genetic architecture, as evidenced by the overlapping GWAS hits between polymorphisms near *TERT* and *TRIM59* that associate with both CHIP and IEAA (Bick, Pirruccello, et al., 2020; Bick, Weinstock, et al., 2020; Zink et al., 2017). However, the heritability of CHIP appears to be low (3.6% Bick, Pirruccello, et al., 2020; Bick, Weinstock, et al., 2020), which limits our ability to test for genetic correlation between CHIP and age acceleration. Previous studies have shown that IEAA of cultured fibroblasts strongly correlates with the number of population doublings (Lu et al., 2018). Therefore, an alternative hypothesis is that the increase in intrinsic age acceleration seen in CHIP carriers may be due to either (1) increased proliferation or self‐renewal of HSC clones that harbor these mutations or (2) stem cell exhaustion of wild‐type HSCs from over‐proliferation, leading to a selective advantage for mutant clones. Studies in model systems such as genetically modified mice may help delineate the cause‐effect relationship between mutations in various CHIP−associated genes and intrinsic age acceleration.

Most importantly, our results show that it is possible to stratify CHIP carriers into those at high versus low risk of adverse clinical outcomes using a composite measure of Hannum and GrimAge (AgeAccelHG). CHIP or AgeAccelHG status alone is associated with a modestly increased risk of death or CHD, but the combination of CHIP+ and AgeAccelHG+ is synergistic for these outcomes. Furthermore, CHIP in the absence of epigenetic aging in these clocks is not associated with adverse outcomes. These results suggest that the effects of CHIP on health are context‐dependent, as Hannum and GrimAge are not uniformly increased in all CHIP carriers, and may be influenced by environmental factors such as CRP, smoking, diet, BMI, insulin resistance, education level, exercise, socioeconomic status (Quach et al., 2017), traumatic stress (Wolf et al., 2018), insomnia (Carroll et al., 2017), and hunter‐gatherer lifestyle (Horvath et al., 2016). Our results may also explain why the strength of the associations between CHIP and mortality or CHD are somewhat inconsistent across studies—while the prevalence of CHIP is fairly uniform across populations, epigenetic aging may not be. In populations with a high prevalence of risk factors for epigenetic aging, the consequences of CHIP may be direr than in populations without such risk factors.

Our risk stratification schema may also be used to select patients for clinical trials of pharmaceutical or behavioral interventions, as the benefit‐to‐risk ratio may be particularly favorable in the high‐risk CHIP group. We note that that the 5‐year mortality after CHD in those who are CHIP+ and AgeAccelHG+ approaches 60%, similar to the mortality seen in patients with intermediate‐risk MDS (Greenberg et al., 2012). Furthermore, the high event rate in this group would enable smaller trials with sufficient power for detecting favorable outcomes such as reduced all‐cause mortality or time to CHD. One such intervention may be blockade of IL‐6 receptor (Bick, Pirruccello, et al., 2020; Bick, Weinstock, et al., 2020); our results show that those who are *TET2*‐CHIP+ and AgeAccelHG+ have lower risk of death or CHD with increasing copies of rs2228145, which has previously been linked to reduced IL‐6R expression levels in myeloid cells (Bick, Pirruccello, et al., 2020; Bick, Weinstock, et al., 2020). Alternatively, this group may benefit from IL‐1B inflammatory blockade (Ridker et al., 2017), which has also been shown to be relevant to atherosclerosis in model systems of CHIP (Fuster et al., 2017; Jaiswal et al., 2017). Of note, AgeAccelHG appears to be superior to hs‐CRP and genotype at *IL6R* for risk discrimination of CHIP carriers, implying that it is capturing additional information beyond baseline inflammation.

In sum, our results show that there is an important relationship between CHIP and epigenetic aging. CHIP and epigenetic age acceleration can also be used to identify persons at high risk of all‐cause mortality and CHD, further reinforcing the importance of both phenotypes as valuable tools in precision medicine for aging.

## 4 METHODS

### 4.1 Epidemiological cohorts

All participant data were obtained from four independent patient cohorts: the FHS (Feinleib et al., 1975), the JHS (Sempos et al., 1999), the WHI (phs000200.v11.p3), and the MESA (Bild, 2002, p. 200). These cohorts were included in the TOPMed consortium which is run by the National Heart Lung and Blood Institute of the National Institutes of Health. Access to all data was approved by TOPMed as well as the individual cohorts. We included only those persons from these cohorts in which the age at draw for both whole blood methylation and WGS were available. In the FHS and JHS cohorts, the samples for methylation and WGS were taken from the same blood draw in all persons. In MESA, methylation data were only used from the first exam as this was the time at which DNA for WGS was also collected. In the WHI cohort, the two samples were often taken from different times. We only considered persons for whom the methylation and WGS samples were taken within 3 years of each other.

### 4.2 Methylation array data

Whole blood methylation was quantified using the Illumina MethylationEPIC or HumanMethylation450k array. Normalized methylation data were submitted to the online methylation clock tool (https://dnamage.genetics.ucla.edu/new) which generates methylation age estimates for seven different clocks: DNAmAge (Horvath, 2013), DNAmHannum (Hannum et al., 2013), DNAmPhenoAge (Levine et al., 2018), DNAmSkinClock (Horvath et al., 2018), DNAmGrimAge (Lu, Quach, et al., 2019), intrinsic epigenetic age acceleration (IEAA) (Lu et al., 2018), and extrinsic epigenetic age acceleration (EEAA) (Lu et al., 2018). Age acceleration was computed for each measure as the residual of model predicting each persons' methylation age from their chronological age at the time of blood draw. Additionally, the DNAmGrimAge clock generates seven surrogate biomarkers based upon blood protein expression (MADM/NRBP1, B2 M, CST3 (Cystatin C), GDF15, LEP (Leptin), SERPINE1/PAI1, and TIMP1) as well smoking pack years. Age‐adjusted LTL and unadjusted LTL are also estimated by the clock software (Lu, Seeboth, et al., 2019). Cell composition was also estimated by the clock software using a published model (Houseman et al., 2012).

### 4.3 Identification of somatic variants

Approximately 100,000 whole genomes were sequenced from whole blood DNA to ~30× depth as part of the TOPMed study (Bick, Pirruccello, et al., 2020; Bick, Weinstock, et al., 2020). Somatic mutations associated with CHIP were called from WGS data using the Mutect2 module in GATK from BAM files previously aligned with BWA. Candidate CHIP variants were selected based upon a curated list of known variants recurrently mutated in hematological malignancies as previously described (Jaiswal et al., 2017) (see Table S6). A full list of variants identified in this study are included in Table S7.

### 4.4 Association between CHIP and methylation age acceleration

Clonal hematopoiesis of indeterminate potential status was associated with age acceleration and the other measures using linear modeling, with a separate model being fitted for each aging measure. Because of the relatively small number of comparisons, *p*‐values for these analyses were reported unadjusted. We combined the data from all three studies and used residualization to remove the effects of age, race/ethnicity, sex, and study. This approach was chosen to eliminate any possibility of spurious associations between CHIP and the methylation measures that were driven by collinearity between CHIP and covariates. The residualized methylation measure was the outcome in each model, and a likelihood ratio test was performed to test the significance of CHIP as predictor against a null model containing only the intercept. When testing the association of CHIP mutations with specific genes, CHIP status was replaced with a categorical variable indicating whether the individual had a mutation in that gene, and persons with CHIP mutations in other genes were excluded. The following specific categories for single mutations were used: *DNMT3A*, *TET2*, DNA‐damage response (DDR, which includes *TP53*, *PPM1D*, and *BRCC3*), *JAK2*, *ASXL1*/*2* (includes *ASXL1* and *ASXL2*), splicing factor (includes *SF3B1*, *SRSF2*, *U2AF1*, *ZRSR2*, and *PRPF8*), and other for any single gene which did not fit in the previous categories. Persons with mutations in more than one gene were classified as multiple regardless of the number of mutations or which genes were mutated, while persons with multiple mutations in the same gene were classified as singletons. The analysis of mutation number versus methylation measures grouped all persons with single mutation into one group, and split the group with mutations in multiple genes into two mutations and greater than two mutations, regardless of which genes were mutated. Correlation between VAF and the residualized methylation measures was computed using biweight midcorrelation, an outlier resistant alternative to Pearson's correlation (Horvath, 2011).

### 4.5 Differential methylation of clock CpGs

Illumina HumanMethylation450K and MethylationEPIC CpG probe IDs for the clocks and DNAmLTL were obtained from the supplemental data of the relevant publications. Methylation beta values for each cohort were subsetted for CpGs used in all clocks except GrimAge (for which the CpG locations have not been published) and were converted to *M*‐values. The *M*‐values were adjusted for the same covariates that were considered for the methylation clock measures. The adjusted residuals were tested for differential methylation and *p*‐values were corrected for the number of CpGs tested using limma(Ritchie et al., 2015).

### 4.6 Association of CHIP and epigenetic age acceleration with clinical outcomes

We tested the associations of CHIP and epigenetic age acceleration with all‐cause mortality and incident CHD with Cox proportional hazards models using the *survival* package in R. Models included age, sex, race/ethnicity, systolic blood pressure, type 2 diabetes status, plasma LDL‐cholesterol concentration, plasma HDL‐cholesterol concentration, plasma triglyceride concentration, and smoking status as covariates. Some persons in WHI had DNA for the methylation and/or WGS sample obtained several years after the baseline visit, which potentially could introduce survivorship bias into the analysis. For this reason, we also excluded anyone in WHI for whom either the methylation or WGS blood draw occurred more than 5 years after the baseline visit.

For analysis of all‐cause mortality, pooled data from FHS, JHS, and WHI EMPC were used. The selection of samples used in TOPMed in these cohorts were taken essentially at random from the larger parent cohorts. WHI BA23 was excluded from this analysis because persons in this cohort were over‐sampled for CHD. MESA was excluded from this analysis because persons in this cohort were selected for surviving at least 10 years from baseline. We chose to present the results from models in which all three cohorts were pooled, rather than analyzed separately and then meta‐analyzed. The results for the meta‐analysis were similar, however (CHIP/AgeAccelHG interaction pooled: coefficient = 0.80, *p* < 3.7 × 10^−3^; CHIP/AgeAccelHG interaction in fixed‐effects meta‐analysis: coefficient = 0.85, *p* < 2.4 × 10^−3^).

For the analysis of CHD, the WHI BA23 cohort was analyzed separately, and a meta‐analysis was used to combine the results of the BA23 analysis with the other pooled cohorts (JHS, FHS, and WHI EMPC) to get the final effect size estimates. 45 persons in WHI BA23 were also included in the mortality analysis of WHI EMPC but were not included in the CHD analysis of WHI EMPC (i.e., were not double‐counted). Because BA23 was over‐sampled for CHD, we adjusted the sample weights in BA23 using race and incident CHD numbers in the entire dbGaP‐eligible set of WHI to allow for Cox proportional hazards modeling. Robust standard errors were used to calculate *p*‐values in all models.

Similar to the associations between CHIP and age acceleration, *p*‐values for these analyses were reported unadjusted due to the small number of comparisons. We used the age acceleration residuals from the analysis associating CHIP with epigenetic age acceleration to determine if persons had high age acceleration (AgeAccelHG, defined as being greater than 0 for both AgeAccelHannum and AgeAccelGrim) and intersected this with CHIP status, resulting in four groups: no CHIP with low age acceleration, no CHIP with high age acceleration, CHIP with low age acceleration, and CHIP with high age acceleration. When we analyzed the interaction of individual clocks with CHIP status, we used the same definition for age acceleration but restricted it to only one clock.

For the gene‐level analyses, persons with any singleton *DNMT3A*, *TET2*, or DDR gene (*TP53*, *PPM1D*, *BRCC3*) mutation were considered to be in those classes. All other non‐*DNMT3A*, *TET2*, and DDR mutations were considered “other.” In those with multiple mutations, the mutated gene with the highest VAF was used to assign the class.

For the analysis of cumulative incidence of death and CHD, the *cmprsk* package in R was used.

## CONFLICT OF INTEREST

S. Jaiswal is a scientific advisor to Novartis, Roche Genentech, and Foresite Labs. UC Regents (the employer of S. Horvath and A. T. Lu) has filed patents surrounding several epigenetic biomarkers of aging (including GrimAge) which list S. Horvath and A. T. Lu as inventors. P. Natarajan reports grants support from Amgen, Apple, and Boston Scientific, and is a scientific advisor to Apple. S. Kathiresan is an employee of Verve Therapeutics and holds equity in Verve Therapeutics, Maze Therapeutics, Catabasis, and San Therapeutics. He is a member of the scientific advisory boards for Regeneron Genetics Center and Corvidia Therapeutics; he has served as a consultant for Acceleron, Eli Lilly, Novartis, Merck, Novo Nordisk, Novo Ventures, Ionis, Alnylam, Aegerion, Haug Partners, Noble Insights, Leerink Partners, Bayer Healthcare, Illumina, Color Genomics, MedGenome, Quest, and Medscape. G. Abecasis is an employee of Regeneron Pharmaceuticals and owns stock and stock options for Regeneron Pharmaceuticals. S. Jaiswal and S. Kathiresan have jointly filed patents relating to clonal hematopoiesis and atherosclerotic cardiovascular disease.

## AUTHOR CONTRIBUTIONS

DN and SJ performed all statistical analyses and writing of the manuscript and DN created all figures and tables. DN, SJ, AL, AB, JW, CK, TA, AP, JGW, and SH contributed to the study design and interpretation of results. AL, AB, JEM, PD, CK, AR, JGW, and SH provided feedback on the writing of the manuscript. AL, AB, PN, JW, MS, SK, GA, KT, XG, RT, PD, YL, CJ, SR, DVDB, CL, TB, GP, AC, LR, AJ, JM, JEM, PD, CK, TA, DL, JR, AR, EW, JGW, and SH contributed to data acquisition and processing.

## Supporting information

Table S6

Table S7

Supplementary Material

## Data Availability

The data that support the findings of this study are available from Trans‐Omics for Precision Medicine (TOPMed) consortium. Restrictions apply to the availability of these data, which were used under license for this study. Data are available at https://www.nhlbiwgs.org/topmed‐data‐access‐scientific‐community with the permission of TOPMed Data Coordinating Center. All data used in the study are available at the follow DbGaP accessions: Framingham Heart Study (phs000974.v1.p1), Jackson Heart Study (phs000964.v4.p1), Women's Health Initiative (phs001237.v2.p1), Multi‐ethnic Study of Atherosclerosis (phs000209.v13.p3).
