# Supplementary material for: Clonal hematopoiesis associated with epigenetic aging and clinical outcomes
Source: Aging Cell. 2021 May 29;20(6):e13366. doi: 10.1111/acel.13366 (PMC8208788; doi:10.1111/acel.13366)
Supplement: Supplementary file 3 — Supplementary Material [file ACEL-20-e13366-s002.pdf]

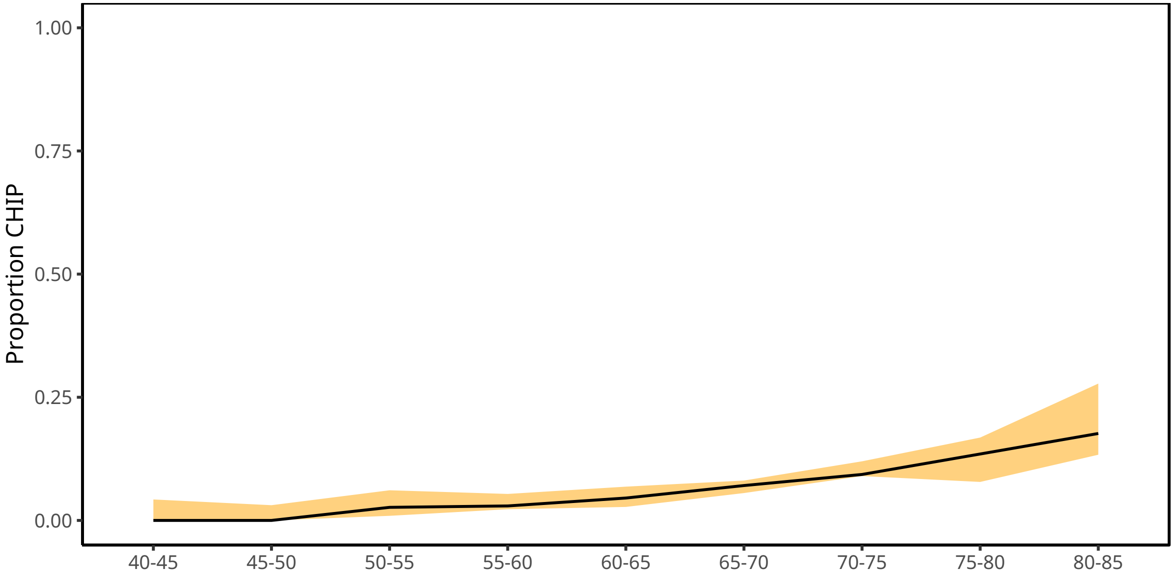

Figure S1. CHIP prevalence increases with age. Plot of percentage of persons with CHIP as a function of age divided in 5 year bins.

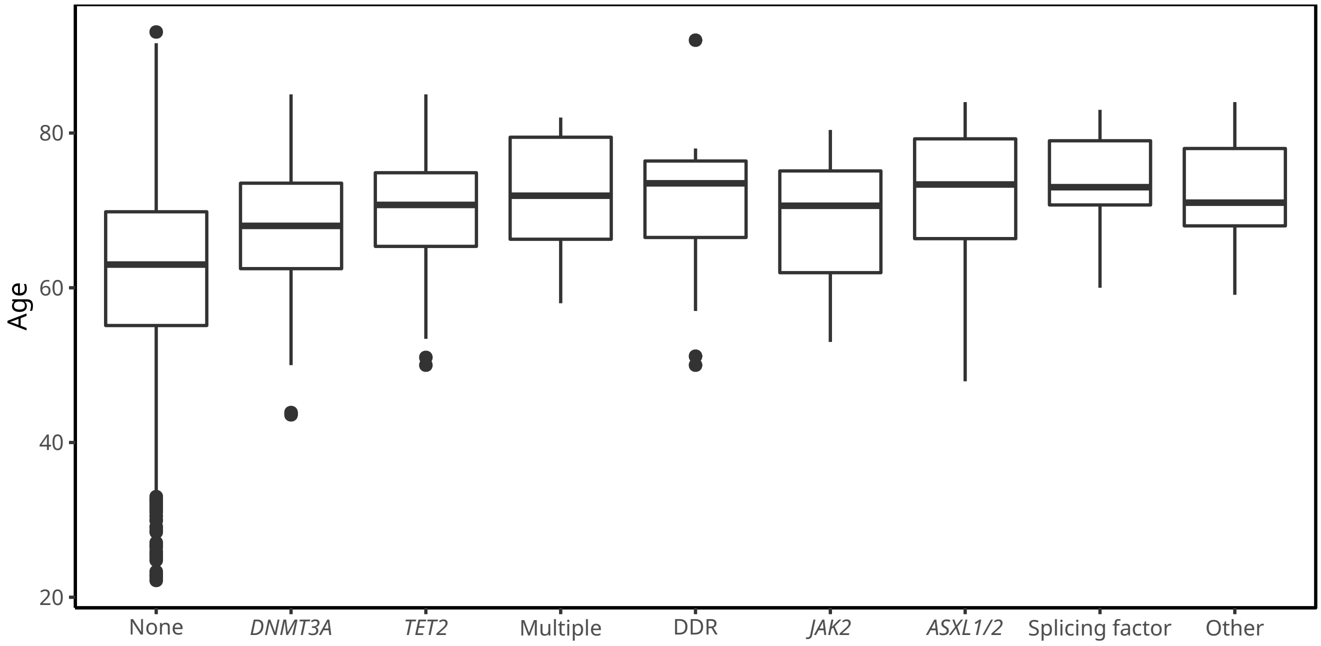

Figure S2. CHIP carriers are older than controls. Boxplot of age of persons with the different classes of CHIP mutations.

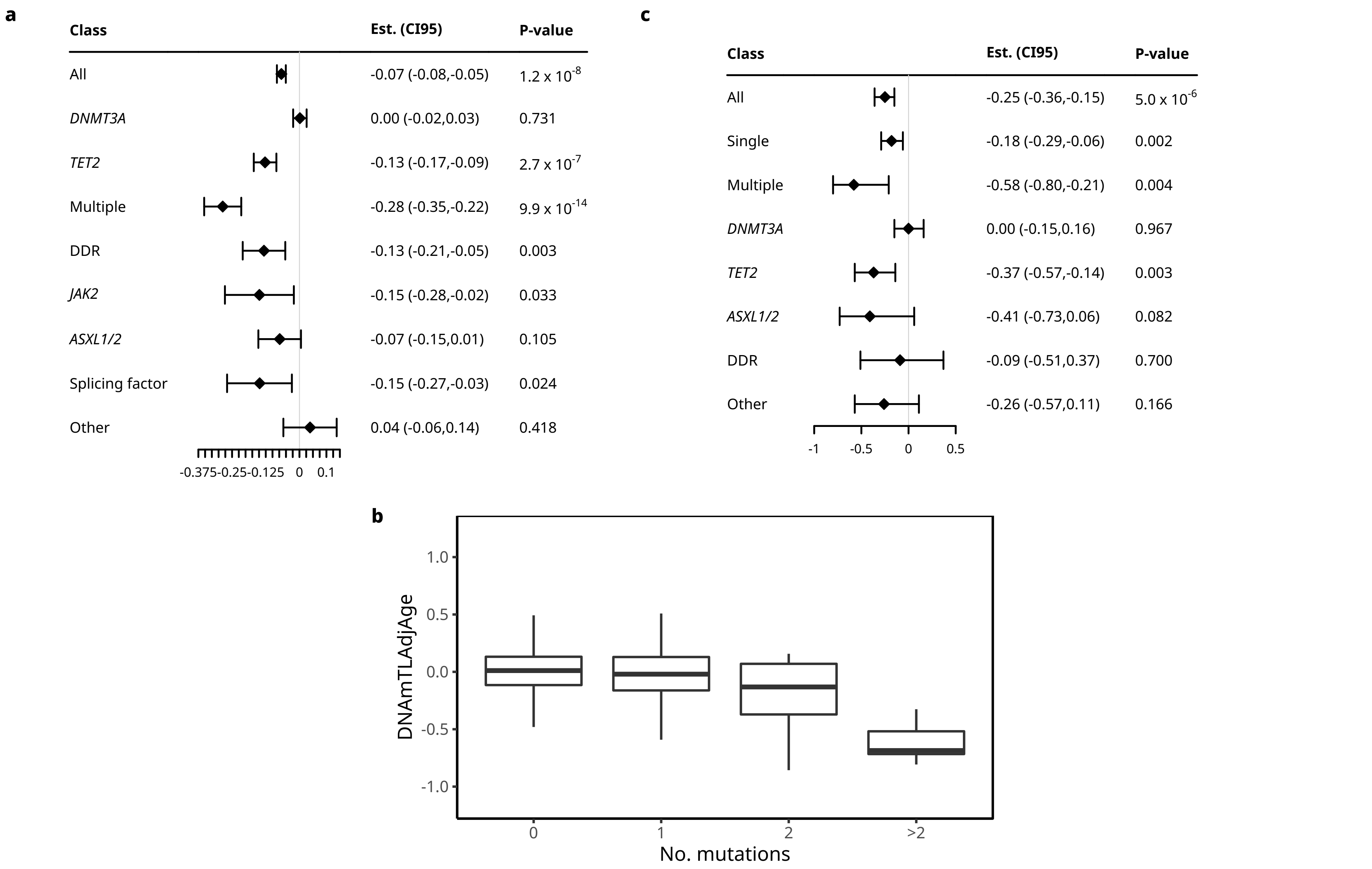

Figure S3. CHIP is associated with decreased methylation-estimated age-adjusted telomere length (DNAmLTLAdjAge). a Forest plot showing confidence intervals and p-values of effects of all CHIP mutations or specific classes of CHIP mutations on DNAmLTLAdjAge. **b** Box plots of DNAmLTLAdjAge as a function of number of CHIP mutations. **c** Forest plot showing confidence intervals and p-values of correlation of variant allele fraction (VAF) with DNAmLTLAdjAge.

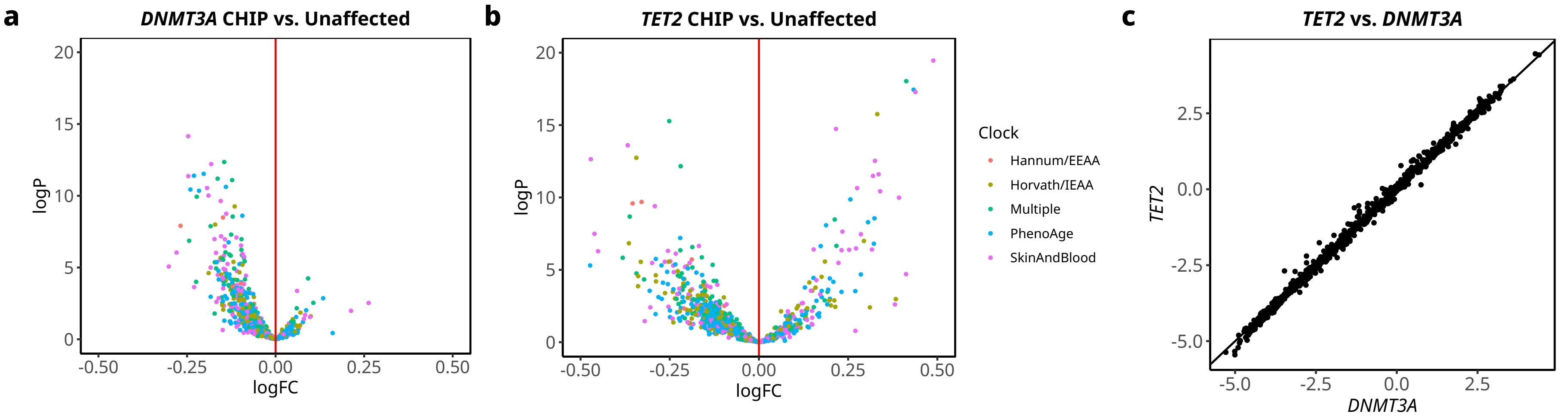

Figure S4. Methylation CpG sites used in clocks are hypomethylated in CHIP carriers. a,b Volcano plots from differential methylation analysis of persons with (**a**) *DNMT3A* and (**b**) *TET2* mutations vs. controls. The x-axis is the log fold change and the y-axis is the -$\log_{10}$ p-value for each CpG. **c** Scatter plot showing the correlation of average M-values at CpGs shown in volcano plots in persons with *DNMT3A* and *TET2* mutations.

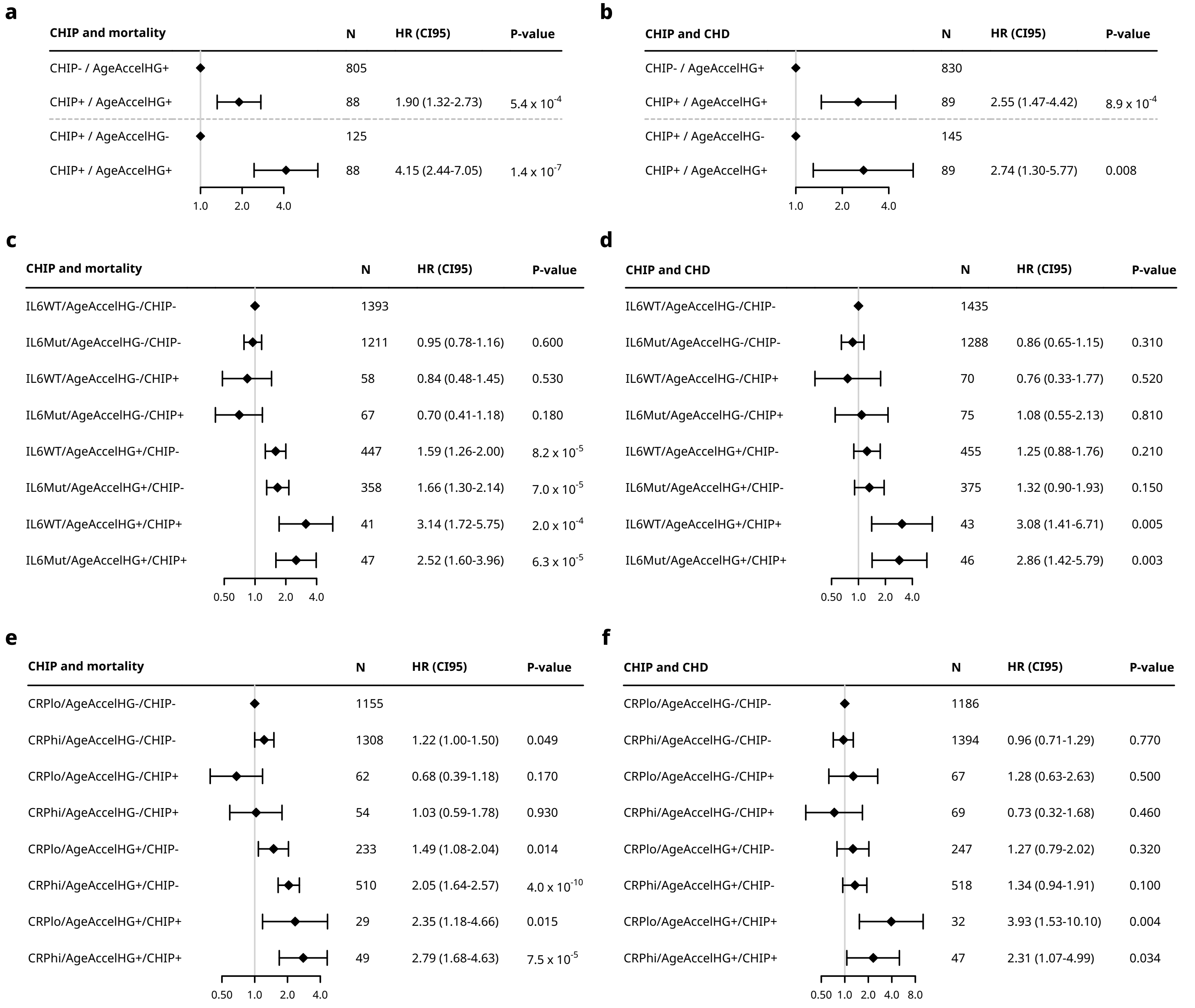

Figure S5. The risk of mortality and coronary heart disease (CHD) increases with CHIP in persons with age acceleration, and with age acceleration in persons with CHIP, and is not affected by rs2228145 genotype or CRP levels. **a,b** Forest plots showing hazard ratios, confidence intervals and p-values for mortality (**a**) and development of CHD (**b**). The top section shows the effect of CHIP in persons with age acceleration, and the bottom section shows the effect of age acceleration in persons with CHIP. **c,d** Forest plots showing hazard ratios, confidence intervals and p-values as a function of CHIP status, age acceleration and rs2228145 genotype for mortality (**c**) and development of CHD (**d**). Persons with at least 1 alternate allele of rs2228145 were designated IL6Mut, and those with no alternate alleles were designated IL6WT. **e,f** Forest plots showing hazard ratios, confidence intervals and p-values as a function of CHIP status, age acceleration and CRP levels for mortality (**e**) and development of CHD (**f**). Persons with greater than 2 mg/L of CRP were designated CRPhi, and those with 2mg/L or less of CRP were designated CRPlo.

CHIP and mortality	N	HR (CI95)	P-value
IL6WT/AgeAccelHG-/TET2_CHIP-	1304		
IL6Mut/AgeAccelHG-/TET2_CHIP-	1117	0.95 (0.80-1.11)	0.510
IL6WT/AgeAccelHG-/TET2_CHIP+	14	0.79 (0.33-1.87)	0.590
IL6Mut/AgeAccelHG-/TET2_CHIP+	9	1.64 (0.74-3.67)	0.230
IL6WT/AgeAccelHG+/TET2_CHIP-	404	1.49 (1.23-1.80)	5.6×10^{-5}
IL6Mut/AgeAccelHG+/TET2_CHIP-	312	1.62 (1.31-2.00)	8.6×10^{-6}
IL6WT/AgeAccelHG+/TET2_CHIP+	8	11.32 (6.85-18.68)	2.4×10^{-21}
IL6Mut/AgeAccelHG+/TET2_CHIP+	8	1.91 (0.96-3.80)	0.066

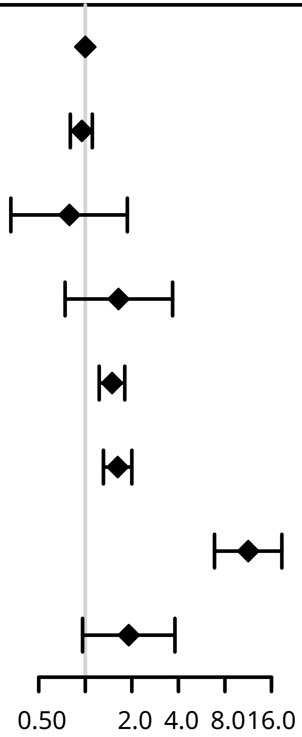

Figure S6. rs2228145 reduces the increased risk in composite mortality/CHD cumulative risk in persons with TET2 CHIP mutations and epigenetic age acceleration. Forest plot showing hazard ratios, confidence intervals and p-values for Cox proportional hazard models of the composite measure of mortality/CHD risk in persons from FHS, JHS, and WHI AS315. Model includes chronological age, race, low-density lipoprotein cholesterol, high-density lipoprotein cholesterol, triglycerides, systolic blood pressure, type 2 diabetes status and smoking status as covariates. IL6Mut = 1 or 2 copies of alternate allele for rs2228145, IL6WT = 0 copies of alternate allele for rs2228145.

		Subjects			Median age		Used in analysis		
Cohort	Self-reported ancestry	Total	Female	Male	Female	Male	Age Acceleration	Mortality	CHD
WHI AS315	Black/AA	225	225	0	64	N/A			
WHI AS315	Hispanic	56	56	0	64	N/A	Yes	Yes	Yes
WHI AS315	White	697	697	0	66	N/A			
WHI BA23	Black/AA	96	96	0	64	N/A			
WHI BA23	Hispanic	27	27	0	67	N/A	Yes	No[1]	Yes[2]
WHI BA23	White	378	378	0	69	N/A			
FHS	Black/AA	2	2	0	74	N/A			
FHS	Hispanic	6	2	4	70	78	Yes	Yes	Yes
FHS	Multiple	8	5	3	66	63			
FHS	White	1365	743	622	67	68			
JHS	Black/AA	1727	1083	644	58	56	Yes	Yes	Yes
MESA	Asian	71	33	38	62	58			
MESA	Black/AA	182	105	77	61	60	Yes	No[3]	No[3]
MESA	Hispanic	287	159	128	58	56			
MESA	White	395	198	197	62	59			

Table S1. Demographics of cohorts used in study. WHI = Women's Health Initiative, FHS = Framingham Heart Study, JHS = Jackson Heart Study, MESA = Multiethnic Study of Atherosclerosis, AA = African American, CHD = coronary heart disease. [1]WHI BA23 was excluded from the mortality analysis because it was a case-control study for CHD. [2]WHI BA23 was analyzed for CHD risk separately from the other cohorts becuase it was a case-control study for CHD. [3]MESA subjects were excluded from mortality and CHD analysis because they were selected for survival over 10 years, which biased subject selection.

a

Class	2 mutations vs. 1 est. (SE)	2 mutations vs. 1 p-value	>2 mutations vs. 2 est. (SE)	>2 mutations vs. 2 p-value
Horvath	2.66 (0.87)	0.002	5.33 (2.27)	0.019
IEAA	2.23 (0.83)	0.007	4.31 (2.17)	0.047
Hannum	4.37 (0.81)	6.8×10^{-8}	9.48 (2.11)	7.0×10^{-6}
EEAA	5.08 (1.06)	1.6×10^{-6}	13.30 (2.76)	1.4×10^{-6}
SkinBloodClock	2.64 (0.65)	4.3×10^{-5}	1.32 (1.68)	0.432
PhenoAge	2.42 (1.16)	0.037	0.84 (3.04)	0.782
GrimAge	2.49 (0.80)	0.002	4.69 (2.09)	0.025

b

Class	2 mutations vs. 1 est. (SE)	2 mutations vs. 1 p-value	>2 mutations vs. 2 est. (SE)	>2 mutations vs. 2 p-value
DNAmADM	0.03 (0.01)	0.007	0.00 (0.03)	0.917
DNAmB2M	0.02 (0.02)	0.170	0.11 (0.05)	0.020
DNAmCystatinC	0.05 (0.01)	1.4×10^{-5}	0.07 (0.03)	0.023
DNAmGDF15	0.06 (0.04)	0.169	0.19 (0.10)	0.064
DNAmLeptin	-0.01 (0.08)	0.886	-0.18 (0.20)	0.384
DNAmPACKYRS	1.95 (2.01)	0.332	5.97 (5.25)	0.255
DNAmPAI1	0.01 (0.04)	0.887	0.10 (0.11)	0.328
DNAmTIMP1	0.02 (0.01)	7.6×10^{-4}	0.01 (0.02)	0.353

c

Class	2 mutations vs. 1 est. (SE)	2 mutations vs. 1 p-value	>2 mutations vs. 2 est. (SE)	>2 mutations vs. 2 p-value
Plasmablast	0.04 (0.04)	0.295	0.01 (0.11)	0.919
Exhausted CD8+ T-cell	-0.03 (0.71)	0.964	6.17 (1.84)	8.1×10^{-4}
Naive CD8+ T-cell	-12.9 (8.5)	0.130	-54.7 (22.3)	0.014
Naive CD4+ T-cell	-31.6 (21.8)	0.148	-2.5 (56.9)	0.966
Natural killer cell	0.00 (0.01)	0.700	0.05 (0.02)	0.020
Monocyte	0.02 (0.01)	0.002	0.07 (0.01)	6.8×10^{-7}
Granulocyte	0.02 (0.02)	0.437	-0.12 (0.06)	0.031

Table S2. Increasing number of CHIP mutations effects age acceleration and other methylation measures. Tables with effect sizes, standard errors and p-values of the comparison of persons with 2 CHIP mutations vs 1, and >2 mutations vs. 2 in (**a**) age acceleration in methylation clocks, (**b**) DNAmGrimAge biomarkers, and (**c**) predicted cell type abundance.

a

Class	DNAmADM estimate (SE)	DNAmADM p-value	DNAmB2M estimate (SE)	DNAmB2M p-value	DNAmCystatinC estimate (SE)	DNAmCystatinC p-value	DNAmGDF15 estimate (SE)	DNAmGDF15 p-value	DNAmLeptin estimate (SE)	DNAmLeptin p-value	DNAmPACKYRS estimate (SE)	DNAmPACKYRS p-value	DNAmPAI1 estimate (SE)	DNAmPAI1 p-value	DNAmTIMP1 estimate (SE)	DNAmTIMP1 p-value
All	0.016 (0.004)	1.0×10^{-4}	0.022 (0.006)	1.9×10^{-4}	0.012 (0.004)	0.002	0.031 (0.013)	0.020	-0.068 (0.024)	0.009	1.735 (0.631)	0.009	0.015 (0.013)	0.274	0.005 (0.002)	0.009
DNMT3A	0.008 (0.006)	0.175	0.004 (0.008)	0.768	-0.005 (0.005)	0.306	-0.007 (0.018)	0.606	-0.028 (0.034)	0.468	1.255 (0.880)	0.189	-0.000 (0.018)	0.962	0.002 (0.003)	0.542
TET2	0.008 (0.009)	0.379	0.032 (0.012)	0.010	0.024 (0.008)	0.004	0.068 (0.027)	0.016	-0.166 (0.053)	0.002	1.445 (1.380)	0.326	0.021 (0.028)	0.481	0.002 (0.004)	0.723
Multiple	0.058 (0.013)	2.2×10^{-5}	0.073 (0.019)	1.7×10^{-4}	0.090 (0.013)	1.2×10^{-12}	0.089 (0.043)	0.042	-0.122 (0.083)	0.154	3.345 (2.150)	0.131	0.030 (0.044)	0.510	0.031 (0.006)	9.5×10^{-7}
DDR	0.014 (0.015)	0.385	0.018 (0.022)	0.446	0.002 (0.014)	0.922	0.089 (0.049)	0.074	0.083 (0.095)	0.360	-1.275 (2.450)	0.577	-0.036 (0.050)	0.460	-0.011 (0.007)	0.112
JAK2	0.050 (0.024)	0.042	0.101 (0.034)	0.004	0.034 (0.022)	0.140	0.119 (0.077)	0.130	0.087 (0.149)	0.542	-0.114 (3.870)	0.957	0.123 (0.079)	0.125	0.031 (0.011)	0.006
ASXL1/2	0.013 (0.015)	0.431	0.034 (0.022)	0.134	0.013 (0.014)	0.386	0.077 (0.049)	0.121	-0.212 (0.095)	0.028	7.535 (2.450)	0.002	0.000 (0.050)	0.989	0.016 (0.007)	0.026
Splicing factor	0.023 (0.023)	0.340	0.035 (0.032)	0.291	-0.013 (0.021)	0.515	0.061 (0.073)	0.411	-0.109 (0.141)	0.458	5.825 (3.650)	0.117	0.127 (0.075)	0.091	-0.013 (0.011)	0.211
Other	0.050 (0.019)	0.009	0.028 (0.027)	0.313	0.019 (0.018)	0.287	-0.035 (0.060)	0.547	-0.032 (0.117)	0.810	-0.071 (3.040)	0.956	0.098 (0.062)	0.118	0.014 (0.009)	0.136

b

Class	Plasmablast estimate (SE)	Plasmablast p-value	Exhausted CD8+ T-cell estimate (SE)	Exhausted CD8+ T-cell p-value	Naive CD8+ T-cell estimate (SE)	Naive CD8+ T-cell p-value	Naive CD4+ T-cell estimate (SE)	Naive CD4+ T-cell p-value	Natural killer cell estimate (SE)	Natural killer cell p-value	Monocyte estimate (SE)	Monocyte p-value	Granulocyte estimate (SE)	Granulocyte p-value
All	0.011 (0.013)	0.424	0.693 (0.222)	0.003	-0.64 (2.68)	0.821	19.15 (6.84)	0.008	-0.002 (0.003)	0.489	0.003 (0.002)	0.156	0.013 (0.007)	0.072
DNMT3A	-0.006 (0.018)	0.696	0.055 (0.309)	0.956	7.87 (3.73)	0.034	24.55 (9.55)	0.014	-0.007 (0.004)	0.066	-0.005 (0.002)	0.030	0.009 (0.010)	0.376
TET2	-0.013 (0.028)	0.629	-0.102 (0.482)	0.772	-2.31 (5.83)	0.697	-7.96 (14.90)	0.546	0.008 (0.006)	0.167	0.012 (0.004)	0.002	-0.006 (0.015)	0.658
Multiple	0.099 (0.044)	0.024	2.968 (0.754)	1.1×10^{-4}	-24.54 (9.12)	0.007	-17.05 (23.30)	0.437	0.001 (0.009)	0.915	0.028 (0.006)	2.2×10^{-6}	0.047 (0.023)	0.046
DDR	-0.007 (0.050)	0.874	2.618 (0.859)	0.003	-16.93 (10.40)	0.103	22.55 (26.60)	0.418	0.020 (0.010)	0.046	-0.001 (0.007)	0.878	-0.008 (0.027)	0.737
JAK2	0.187 (0.079)	0.018	2.268 (1.360)	0.100	-14.13 (16.40)	0.391	34.65 (42.00)	0.423	-0.028 (0.016)	0.081	0.013 (0.011)	0.224	0.132 (0.042)	0.002
ASXL1/2	0.062 (0.050)	0.219	1.688 (0.859)	0.054	-6.48 (10.40)	0.535	63.15 (26.60)	0.019	-0.006 (0.010)	0.579	0.007 (0.007)	0.268	0.016 (0.027)	0.564
Splicing factor	-0.022 (0.074)	0.765	1.328 (1.280)	0.312	-3.85 (15.50)	0.805	90.75 (39.60)	0.023	-0.002 (0.015)	0.892	-0.007 (0.010)	0.444	0.036 (0.040)	0.373
Other	0.026 (0.062)	0.676	1.958 (1.070)	0.071	-4.11 (12.90)	0.752	28.35 (32.90)	0.407	-0.007 (0.012)	0.582	-0.001 (0.008)	0.878	0.024 (0.033)	0.480

Table S3. CHIP mutations in specific classes of genes affect Grim biomarkers and predicted cell type abundances. Tables with effect sizes, standard errors and p-values of the effects of CHIP mutations in specific classes of genes on (**a**) DNAmGrimAge biomarkers and (**b**) predicted cell type abundance. ADM = Adrenomedullin; B2M = Beta-2-Microglobulin; GDF15 = Growth Differentiation Factor 15; PACKYRS = Smoking pack years; PAI1 = Plasminogen Activation Inhibitor 1; TIMP1 = Tissue Inhibitor of Metalloproteinases 1

a

Class	Horvath bicor	Horvath p-value	IEAA bicor	IEAA p-value	Hannum bicor	Hannum p-value	EEAA bicor	EEAA p-value	SkinBloodClock bicor	SkinBloodClock p-value	PhenoAge bicor	PhenoAge p-value	GrimAge bicor	GrimAge p-value
All	0.091	0.109	0.106	0.060	0.198	4.2×10^{-4}	0.213	1.4×10^{-4}	0.129	0.022	0.100	0.076	0.185	9.7×10^{-4}
Single	0.070	0.233	0.086	0.141	0.136	0.020	0.154	0.009	0.078	0.186	0.071	0.225	0.152	0.009
Multiple	0.081	0.713	0.056	0.800	0.519	0.011	0.455	0.029	0.372	0.080	0.159	0.468	0.367	0.085
DNMT3A	0.167	0.035	0.190	0.017	0.110	0.168	0.122	0.126	0.141	0.076	0.212	0.007	0.092	0.247
TET2	0.085	0.509	0.070	0.586	0.306	0.015	0.205	0.107	0.045	0.727	-0.243	0.055	0.240	0.058
ASXL1/2	-0.510	0.026	-0.297	0.216	-0.375	0.114	-0.210	0.387	-0.565	0.012	-0.260	0.282	0.227	0.350
DDR	0.153	0.521	0.147	0.535	0.330	0.155	0.382	0.096	0.312	0.181	0.008	0.973	0.089	0.710
Other	-0.170	0.370	-0.190	0.316	0.061	0.748	0.120	0.527	-0.015	0.936	-0.044	0.816	0.131	0.491

b

Class	DNAmADM bicor	DNAmADM p-value	DNAmB2M bicor	DNAmB2M p-value	DNAmCystatinC bicor	DNAmCystatinC p-value	DNAmGDF15 bicor	DNAmGDF15 p-value	DNAmLeptin bicor	DNAmLeptin p-value	DNAmPACKYRS bicor	DNAmPACKYRS p-value	DNAmPAI1 bicor	DNAmPAI1 p-value	DNAmTIMP1 bicor	DNAmTIMP1 p-value
All	0.152	0.007	0.210	1.7×10^{-4}	0.049	0.386	0.139	0.014	0.027	0.636	0.125	0.027	0.068	0.232	0.096	0.090
Single	0.127	0.030	0.181	0.002	-0.017	0.777	0.122	0.037	0.048	0.413	0.124	0.035	0.053	0.365	0.074	0.209
Multiple	0.254	0.242	0.063	0.774	0.374	0.079	0.650	7.9×10^{-4}	0.296	0.170	-0.151	0.492	0.408	0.053	0.243	0.263
DNMT3A	0.192	0.015	0.069	0.387	-0.022	0.783	0.045	0.571	0.054	0.501	0.064	0.420	0.011	0.887	0.022	0.783
TET2	0.033	0.797	0.254	0.045	-0.024	0.853	0.121	0.344	0.046	0.720	0.238	0.060	0.060	0.641	-0.011	0.929
ASXL1/2	0.071	0.773	0.367	0.123	-0.010	0.969	0.159	0.515	-0.086	0.727	0.201	0.409	0.081	0.743	0.600	0.007
DDR	-0.080	0.739	0.200	0.397	-0.238	0.313	0.029	0.905	0.156	0.512	0.034	0.888	0.018	0.941	-0.085	0.722
Other	0.089	0.639	0.164	0.385	-0.138	0.466	0.256	0.171	0.028	0.882	0.099	0.604	0.247	0.188	0.057	0.763

c

Class	Plasmablast bicor	Plasmablast p-value	Exhausted CD8+ T-cell bicor	Exhausted CD8+ T-cell p-value	Naive CD8+ T-cell bicor	Naive CD8+ T-cell p-value	Naive CD4+ T-cell bicor	Naive CD4+ T-cell p-value	Natural killer bicor	Natural killer p-value	Monocyte bicor	Monocyte p-value	Granulocyte bicor	Granulocyte p-value
All	0.069	0.223	0.154	0.006	-0.111	0.050	-0.014	0.800	-0.002	0.974	0.074	0.190	0.090	0.110
Single	0.052	0.380	0.145	0.013	-0.081	0.169	-0.007	0.903	-0.009	0.885	0.007	0.911	0.084	0.152
Multiple	0.010	0.964	0.159	0.468	-0.119	0.588	-0.031	0.887	0.087	0.694	0.322	0.135	-0.015	0.946
DNMT3A	0.031	0.694	0.102	0.199	-0.049	0.539	0.017	0.828	-0.041	0.605	-0.019	0.811	0.082	0.305
TET2	-0.041	0.748	0.018	0.887	0.037	0.776	-0.302	0.016	0.254	0.045	0.106	0.409	-0.151	0.239
ASXL1/2	-0.008	0.973	0.341	0.154	-0.137	0.577	0.450	0.053	-0.389	0.100	-0.127	0.605	0.463	0.046
DDR	0.112	0.639	0.226	0.338	-0.343	0.138	0.152	0.523	-0.100	0.675	0.138	0.561	0.019	0.937
Other	0.323	0.082	0.055	0.772	-0.069	0.717	0.062	0.744	-0.138	0.466	0.088	0.642	0.405	0.026

Table S4. Variant allele fraction is correlated with some methylation measures. Tables with correlation coefficients, standard errors and p-values of the effects of correlation of VAF in all CHIP persons and in specific classes of genes on (**a**) age acceleration in methylation clocks, (**b**) DNAmGrimAge biomarkers, and (**c**) predicted cell type abundance.

	Horvath		IEAA		Hannum		EEAA		SkinBloodClock		PhenoAge		GrimAge	
Group	HR (SE)	p-value	HR (SE)	p-value	HR (SE)	p-value	HR (SE)	p-value	HR (SE)	p-value	HR (SE)	p-value	HR (SE)	p-value
CHIP+	0.195 (0.20)	0.335	0.373 (0.19)	0.050	-0.202 (0.23)	0.385	0.094 (0.22)	0.664	0.258 (0.21)	0.212	0.289 (0.20)	0.141	-0.154 (0.24)	0.525
AgeAccel+	0.199 (0.08)	0.012	0.150 (0.08)	0.059	0.275 (0.08)	5.3×10^{-4}	0.230 (0.08)	0.004	0.162 (0.08)	0.043	0.416 (0.08)	2.5×10^{-7}	0.583 (0.08)	9.8×10^{-13}
CHIP+/AgeAccel+	0.010 (0.26)	0.968	-0.252 (0.26)	0.325	0.566 (0.28)	0.046	0.171 (0.27)	0.527	-0.066 (0.26)	0.802	-0.146 (0.26)	0.572	0.472 (0.29)	0.103

Table S5. The Hannum and Grim clocks interact with CHIP to increase risk of mortality. Table with hazard ratios, standard errors and p-values of Cox proportional hazard models for the interaction of CHIP and epigenetic age acceleration with mortality for individual clocks.
